# Pathophysiological changes of inflammatory syndrome in multiple sclerosis after instituting therapeutic plasmapheresis

**Published:** 2017

**Authors:** S Vlaic, V Poalelungi, M Bălăeţ, VL Purcărea, C Bălăeţ, BI Coculescu

**Affiliations:** * “Alessandrescu-Rusescu” National Institute for Maternal and Child Health, Bucharest, Romania; ** ”Floreasca” Emergency Hospital, Bucharest, Romania; *** University of Birmingham, United Kingdom; **** “Carol Davila” University of Medicine and Pharmacy, Bucharest, Romania; ***** “Lil Med” Clinic, Bucharest, Romania; ****** Center for Medical-Military Research, Bucharest, Romania; “Titu Maiorescu” University, Faculty of Medicine, Bucharest, Romania

**Keywords:** therapeutic plasmapheresis, multiple sclerosis, leukergia test, total blood viscosity test, blood filterability test

## Abstract

In autoimmune conditions affecting the central and peripheral CNS as well as in multiple sclerosis (MS), the inflammatory syndrome is present with the onset of this disease. The present paper aimed to highlight the inflammatory syndrome based on the leukergia test, the total blood viscosity test, blood filterability test as well as on other tests. The early instituting of the therapeutic plasmapheresis beneficially modified the clinical status, the biological and pathophysiological behavior of the patient’s illness.

**Objective of the paper:** The aim was to highlight the importance, advantages, and pathophysiological changes after therapeutic plasmapheresis in five cases, in patients hospitalized with the diagnosis of multiple sclerosis.

**Material and method:** In order to emphasize the inflammatory syndrome, the determination of leukergia assay, the total blood viscosity test and the blood filterability test were added to regular examinations, conducted on the batch of patients included in the study.

**Results and discussions:** As a result of using therapeutic plasmapheresis, the inflammatory parameters in patients with multiple sclerosis improved beneficially as it was proven by the values of inflammatory tests before and after plasmapheresis.

**Conclusions:** In the treatment of multiple sclerosis, plasmapheresis proved to be a medical method that significantly reduced autoimmune inflammatory “installed” syndrome.

## Introduction

The idea of a method to clean blood from toxic substances belongs to Fleig, who launched it in 1910. In 1914, Abel-Rountree and Turner tried to put in place an artificial kidney. In Romania, the first attempts regarding therapeutic plasmapheresis were performed by Sandu Lucian, MD, in 1972 at Colentina Hospital, and by the university professor Victor Voicu, MD, at the Centre for Medical-Military Research, AIC section of “Floreasca” Emergency Hospital. In the Western Europe and the USA, plasmapheresis has been intensively applied in numerous pathologies since the 1980s. In Romania, with the help of professor Ioan Gerota, after 1990, at the same time with the modernization of blood transfusion, the basis of plasmapheresis was set up as a method of extracting plasma for transfusion, not plasmapheresis as a therapy. 

The Romanian Medical Association for Plasmapheresis was established in 2011, and in 2013, the Ministry of Health, by Order of Ministry of Health no. 850/ 8 July 2013 sanctioned therapeutic emergency plasmapheresis, the costs of which were supported by the Health Insurance Fund. 

As a therapeutic method or as a method for plasma extraction, plasmapheresis can be performed with adequate devices and kits by two procedures: by blood centrifugation or by blood filtration. Extracting a big volume of plasma in a short time (50-600 ml) without affecting the oxiphoric function (restoration of the circulation of red cells) has intensified and developed plasmapheresis as an essential attribute in the therapeutic arsenal of many illnesses, speeding up healing or bringing to normal the functions of organs, of the body physiology as a whole. 

Therapeutic plasmapheresis has a great therapeutic and prophylactic potential as it can be used both in emergencies and in substantive treatments, in intoxications, dyslipidemias, allergies, organ deficiency, autoimmune diseases such as multiple sclerosis, Guillain–Barré syndrome (GBS), systemic lupus erythematosus (SLE), rheumatoid arthritis (RA), etc.

The present paper highlighted the alterations of inflammatory tests after plasmapheresis in 5 cases of multiple sclerosis. 

**Principle of the method**

The membrane of red blood cells (erythrocytes) is made of a double lipid layer, lined on the inner face (cytoplasmic face) with a net of characteristic proteins (spectrin and actin), linked to one or more proteins including glycophorin, which crosses the lipid layer, playing an important role in determining the mechanical characteristics of the red blood cell membrane. In small blood vessels (in the sanguineous vessel organ), the normal red blood cells are analyzed downstream, during the flow (especially in the big vessels) to the central part, becoming longer ellipsoids as the flowing speed is higher [**1**-**6**].

The rheological characteristics of the red blood cell are determined by their shape, their viscous-elastic properties of the membranes, the presence of certain markers of inflammation, and by the physical state of the content (state of the haemoglobin). The shape of a biconcave disc, characteristic for the erythrocytes is the result of the interaction of several factors: surface tension of the membrane, report between the radius of the cell and thickness of the membrane, difference of tensions at the level of the external and internal sides of the membranes, rigidity to extension [**7**,**8**].

The changes of the chemical component of the plasma, pH changes, and inflammations due to any cause, can affect the rheological properties of blood. The variation of the negative electric potential, forming the usual load of the normal erythrocyte can lead to rheological modifications of blood. The emergence of inflammatory proteins will produce bioelectrical modifications and increase the blood viscosity. A part of the inflammatory proteins will dress the erythrocytes resulting in an increase of the ESR, the viscosity and modification of the bio-electricity of blood [**9**,**10**].

According to the protocol, plasmapheresis (exchange of plasma) will “wash” the vascular space and bring to normal the value of the inflammatory tests. The mechanism of “cleansing” is inverse to that of viscosity increase that decreases the inter-cellular distance by adding the inflammatory factors, increasing the value of the capillary hematocrit, seizing increased quantities of plasma [**11**,**12**]. The decrease of viscosity, the important physiopathological factor of any inflammatory disease will lead to an improvement in blood flow especially in the small vessels, recharging the tissues by a better oxygenation, facilitating the exchanges between blood and cells [**1**,**2**,**13**].

## Material and methods 

During the year 2015, a number of 5 patients (3 women and 2 men) between 30 and 38 years old, diagnosed with multiple sclerosis, presented themselves to be admitted for therapeutic conduct. The start of the disease as well as the initial diagnosis was rather recent, 2-3 months.

The clinical examination, paraclinical modifications (MRI) and laboratory tests, established an anti-inflammatory treatment and suggested an inflammatory syndrome of average intensity.

The blood samples collection from all the patients to determine the ESR, the reactive protein C, albumin, total calcium, blood viscosity and filterability, as well as the leukergic state of the polymorphonuclear leukocytes before the plasmapheresis procedures.

The ESR, albumin, reactive C protein, calcium and other biochemical tests were determined following the current lab procedures, and the leukergia, viscosity and filterability tests were performed by our own procedures and homologations.

**Fig. 1:**
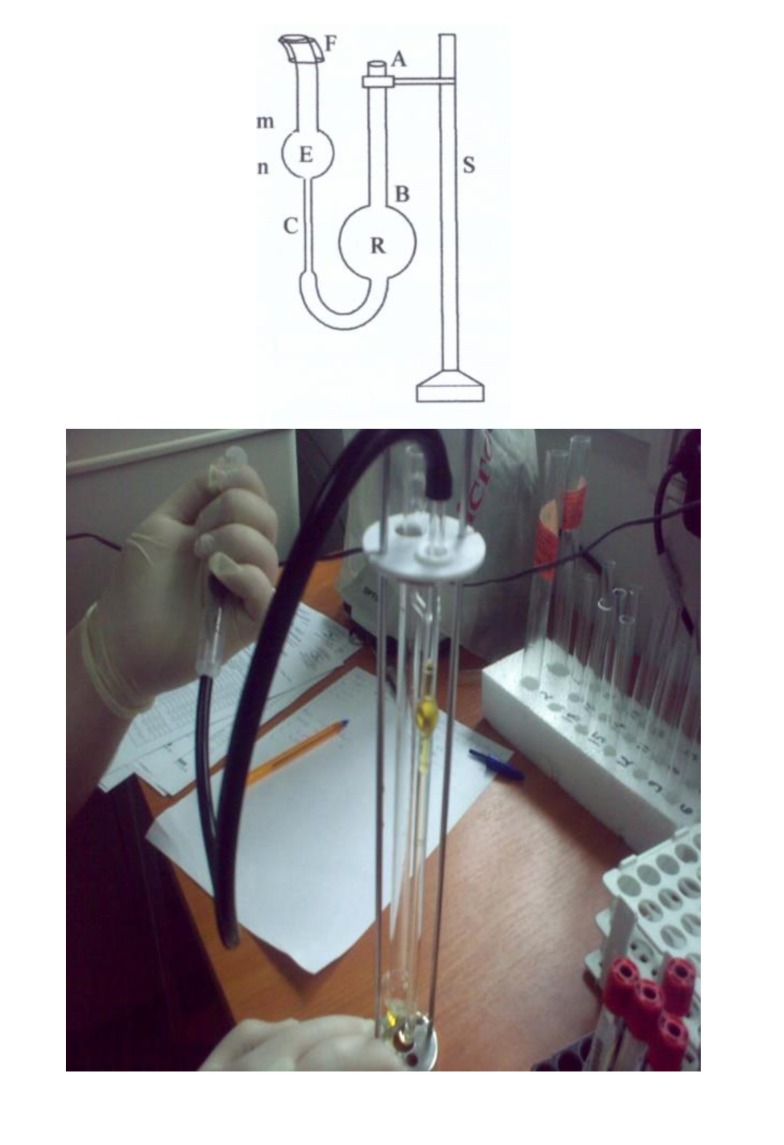
Ostwald viscometer (where S = support, R = big tank, E = small tank, m, n = significant markers, F = rubber tube for aspiration, C = capillary tube).

Beside the specific medicinal treatment, a protocol regarding the plasmapheresis procedures by centrifugation was established.

Five plasmapheresis procedures were performed on each patient, at a 48 hours interval. After each procedure, blood was collected to determine the above-mentioned inflammatory parameters. After the second, third, fourth and fifth procedure, normal saline solution, and electrolytes, the equivalent of the extracted plasma (600 ml) was introduced into the vascular layers of the patients.

The viscosity test was performed with the Ostwald viscometer (**Fig. 1**). The viscosity meter is technically conformable to standard 3105, ASTM 446 and D 2515.

Ostwald viscometer is based on Hagen-Poiseuille law: QV = R4 ∆p/ 8ȵ. The law shows that the flow rate of a fluid running through a cylindrical pipeline is commensurate with the radius of the pipeline raised to the fourth and with the pressures applied on the two ends of the pipeline, and in an inverse ratio to the length of the pipeline and the viscosity of the liquid. 

In order to perform the testing, an aspiration pump attached to the rubber tube and a chronometer was further needed. The viscosity meter used was cleaned in order to eliminate any trace of dust. Calibration was performed with distilled water. 

The temperature in the laboratory was invariable during the experiments (18-20oC). Blood was collected in the vacutainer tube for blood counts and it was homogenized by repeated slight bent. Blood was aspirated into a Pasteur dropper made of plastic, and then it was introduced in tank R through A. Using the rubber aspiration pump applied in F, blood was aspirated from tank R into the small tank E, until the level of the liquid overpassed m by 1-2 cm. The rubber tube was let free, which started the flow of the fluid. At the moment the blood meniscus reached the marker (m), the chronometer started. That was stopped when the meniscus reached the marker (n). The reading on the chronometer represented the duration of the flow (t). Several determinations were performed for each blood sample, calculating an average of the viscosities.

Viscosity was calculated by the formula: U=K (t-v), where U = viscosity, K = constant of the viscosity meter, t = period of time when the liquid covered the distance between m and n, v = correction of the kinetic energy.

Normal values of viscosity for total blood: 3,5-5,4 cp, for plasma: 1,9-2,3 cp, and for serum: 1,6-2,2 cp (where cp = centipoises).

The filterability test was performed as it follows: the blood collected in the vacutainer tube for the blood count after the viscosity test was filtered through a filter paper of 2-5 microns porosity, writing down the filtering time of the total quantity of blood in the vacutainer.

Normal values for filterability: whole blood collected on anticoagulant: 12-16 minutes, blood washed < 12 minutes.

Leukergia test. A method to highlight the leukergic state of the polymorphonuclear leukocytes was performed and homologated by Dr. Bălăeț in the lab of Lil Med Clinic. For each patient in the studied group, a vein blood smear was performed, collected on blood thinner, in a vacutainer tube for blood count. After half an hour, the vacutainer tube with the blood sample was slightly homogenized, and then with a Pasteur dropper, a small quantity of blood was collected, letting 2-3 drops slide (one next to another) on a glass lamella at 45 degrees. The blood that covered the surface of the entire lamella was left to dry at room temperature, then it was cooled to -5ºC in order to achieve erythrocyte (fast freezing and defreezing), a technique that did not affect the granulocytes. The smear was fixed by methanol solution, then the surplus was removed, and at the end, hematoxylin was added, keeping it for 5 more minutes. After those operations, the lamella with the smear was slightly rinsed with distilled water and put to dry at room temperature, and then it was read on the immersion microscope in the same manner as the May-Grunwald-Giemsa colored smear.

The test was positive if during the examination on several microscopic fields there were granulocytes grouped by three, four, five, the distance between the nuclei being smaller than the diameter of a cell. 

## Results

On hospital admission, the ESR in every case was between 28 and 35 mm, and the C protein, reactive positive. 

The average of the results of the tests on the five cases admitted with the diagnosis of multiple sclerosis is presented in the table below. 

**Table 1:** Monitoring of the disease activity and efficiency of the plasmapheresis evaluation in the dynamics of blood markers of the inflammatory syndrome of multiple sclerosis

Tests	ESR mm	Test of filterability	Test of leukergia	Test of viscosity (p)	Protein C reactive	Observations on procedure
Before plasmapheresis	35	18 min.	positive	5,6	positive	
After procedure I	35	18 min.	positive	5,6	positive	at 48 h
After procedure II	30	17min.	positive	5,5	positive	at 48 h
After procedure III	30	17 min.	positive	5,4	positive	at 48 h
After procedure VI	20	16 min.	negative	5,4	negative	at 48 h
After procedure V	15	15 min.	negative	5	negative	

No significant modifications of total calcium in blood, electrolytes, and albumin were noticed. The specific medicinal treatment was administered for the whole duration of the procedures.

## Discussions

The modifications that occurred in 5 patients (3 women and 2 men) diagnosed with multiple sclerosis, were monitored before and after the plasmapheresis protocol. Beside the usual laboratory inflammatory tests (protein C, fibrinogen, ESR), 3 tests were performed: that of total blood viscosity, filterability, and leukergia [**2**-**4**]. Before plasmapheresis, the inflammatory tests (ESR, protein C) emphasized an inflammatory syndrome of medium intensity in our opinion, and correlated to those of total blood filterability, total blood viscosity, the latter being slightly increased and the test of leukergia was positive [**3**]. All the investigated values in the present study became normal after the fourth plasmapheresis procedure. This was what made the neurologist readjust the drug doses, decreasing them by almost 1/ 3 [**2**].

In order to evaluate the intensity of the inflammatory syndrome, 3 lab investigations less used (viscosity, filterability and blood leukergia) were performed because we considered the deformation capacity of erythrocytes as a vascular component modifying in inflammations the blood viscosity as an important marker seen in all inflammatory syndromes. Another important marker in cells is leukergia [**4**,**10**]. The latter means the modification of neutrophil granulocytes in the presence of an antigen that attracts them by chemotaxis thus they become a pile [**3**-**13**], they aggregate, become more mobile, have a more viscous cytoplasm, and a higher capacity to fagocitate. 

## Conclusions

Therapeutic plasmapheresis is an important procedure in multiple sclerosis, together with a specific anti-inflammatory treatment.

Therapeutic success depends on starting the procedure of plasmapheresis as close as possible to the beginning of the illness. 

**Authors Contributions**

The authors equally contributed to this work. All authors read and approved the full paper.
